# Changes in Technology Acceptance of Smart Care Beds Among Long-Term Care Workers in Korea

**DOI:** 10.3390/healthcare12212195

**Published:** 2024-11-04

**Authors:** Young-Sun Kim, Hyeri Shin, Minah Lee, Nam-Hwa Kim, Eui-Hyun Kim, Dukyoung Jung, Minra Choi, Kyeong-Hee Choi

**Affiliations:** 1 Department of Gerontology, AgeTech-Service Convergence Major, Kyung Hee University, Yongin-si 17104, Gyeonggi-do, Republic of Korea; zisoa@khu.ac.kr (H.S.); minah.rhee@khu.ac.kr (M.L.); knwnt712@khu.ac.kr (N.-H.K.); euihyun@khu.ac.kr (E.-H.K.); 2 Industry-University Cooperation, Eulji University in Korea 221, Yatap-dong, Bundang-gu, Seongnam-si 13503, Gyeonggi-do, Republic of Korea; dyjung@eulji.ac.kr (D.J.); 20240929@eulji.ac.kr (M.C.); 3 Regional Industrial Innovation Department (ESH), Research Institute of Sustainable Development Technology, Korea Institute of Industrial Technology, Cheonan-si 31056, Chungcheongnam-do, Republic of Korea; ckhee@kitech.re.kr

**Keywords:** technology acceptance, long-term care workers, smart care bed, empirical study, assistive care devices

## Abstract

Objectives: This study investigates the changing perceptions of Korean care workers regarding a robotic bed designed to assist with repositioning and prevent pressure ulcers. With the primary aim of assessing the technology acceptance among care workers using the robotic bed to solve the problem of a shortage of care workers in Korea, we sought to examine the possibility of applying care robots in the field. Methods: A total of 20 long-term care workers participated in the experiment, and their attitudes were measured before and after using the robot. Frequency analysis and paired *t*-tests were conducted using Stata 17 to analyze the data. Results: The results show significant changes in the perceived ease of use (PEOU), facilitating conditions (FCs), and gerontology anxiety (GA), with the PEOU increasing by 19.87%, FC increasing by 20.63%, and GA decreasing by 17.2%. However, there was no significant change in the perceived usefulness (PU) and intention to use (IU). Conclusions: The results showing that the perception of technology acceptance changed significantly mean that the use of the care robot means that there is a high possibility of positive perceptions in Korean care settings when care robots are distributed in the field in the future, considering that the experimental environment was limited due to the early stage of development of care robots. This study highlights the need for practical demonstrations and thorough training to improve technology acceptance among care workers before the application of care technology in the long-term care environment in South Korea.

## 1. Introduction

The aging population in South Korea has rapidly increased the demand for older adult care services. In response, long-term care insurance for older adults was implemented in 2008 and has since gradually expanded its coverage, with 10.7% of individuals aged 65 and above eligible for long-term care services by the end of 2022 [1]. However, concerns persist regarding a potential shortage of the workers required to address this growing demand. Notably, a high percentage of the older adult care workforce in Korea are older individuals themselves, particularly care workers. According to the 2022 Long-Term Care Survey, 62.9% of care workers are aged 60 and older [2]. Furthermore, an OECD report projects that South Korea will encounter a severe shortage of care workers by 2040, with a key contributing factor being the physically demanding nature of care work [3]. Tasks such as patient transfers, bathing, repositioning, feeding, and assisting with toileting require substantial physical exertion and carry a high risk of musculoskeletal pain and injuries, particularly among the predominantly middle-aged and older female caregivers [4,5]. As per the 2022 Long-Term Care Survey, 15% of care workers and 14.1% of nurses (including nurse aides) reported experiencing musculoskeletal disorders.

In response to the growing need to alleviate the physical strain on care workers, as well as to address gaps in care, discussions have been ongoing regarding the potential introduction of care robots. Since 2019, the Korean government has initiated research and development projects that aim to create care robots specifically tailored for older adults and people with disabilities. The primary goal of these projects is to reduce the occurrence of musculoskeletal disorders and mitigate the physical burden placed on care workers. Notably, ongoing research initiatives focused on the development and enhancement of transfer-assist robots are among these endeavors. These robots are specifically designed to aid in the physically demanding task of transferring patients, which is known to contribute to musculoskeletal pain and injuries among care workers. The Ministry of Trade, Industry, and Energy operated the “Phase 1 Demonstration and Advancement of Transfer-Assist Robots” in 2019–2022 and is currently continuing the Phase 2 research and development project under the same name for 2023–2024. Furthermore, the Ministry of Health and Welfare has been engaging in a two-phase project for “Nine types of care robot development and test” in 2010–2013 and 2024–2027, respectively. Considering these developments, the introduction and integration of new technologies such as care robots into caregiving practices necessitate a thorough consideration of the factors influencing technology acceptance among workers. Actual aged care tasks are often physically demanding and time-constrained, which can hinder the effective utilization of care robots if care workers do not fully accept the technology. In this context, this study aims to highlight the importance of technology acceptance concerning care robots, such as smart care beds, particularly in light of the high average age and work characteristics of Korean care workers. Therefore, the examination of technology acceptance among care workers, who serve as the primary users of these robotic systems, must be prioritized.

Pressure ulcer management is one critical task for caregivers. Although pressure ulcer prevention mattresses and air mattresses are currently used, the reported issues with their design prevent complete pressure ulcer prevention [6]. Consequently, to manage pressure ulcers, caregivers must still perform rounds every two hours to reposition bedridden patients, leading to a significant workload and increased risk of injury among caregivers.

Therefore, this study aims to empirically evaluate and measure technology acceptance among care workers working in long-term facilities using a robotic bed for repositioning older adults. The technology acceptance model (TAM) is a theoretical framework developed based on the theory of reasoned action [7] and the theory of planned behavior [8], which explains that the perceived usefulness and perceived ease of use of new technologies or information systems are shaped by various external factors. In turn, the perceived usefulness and ease of use influence attitudes toward technology and intentions to use it, ultimately affecting its acceptance and usage [9].

Perceived usefulness (PU) refers to the degree to which a person believes that using an information system will enhance their work efficiency [10]. In other words, it can be interpreted as the belief that using smart care beds will reduce the workload and improve the work efficiency of caregivers. Research on the intention to use assistive robots has shown that higher perceived usefulness is associated with a higher intention to use them [10,11,12,13,14].

Perceived ease of use (PEOU) means that using the information system or technology requires little cognitive effort. If users experience discomfort during the use of technology, they may perceive it as being less useful, which could reduce their intentions to use it [15]. Numerous studies have also shown that perceived ease of use positively influences use intentions [13,16,17,18].

Attitude (AT) is defined as an individual’s overall evaluation of performing a particular behavior. According to the theory of reasoned action, people’s attitudes toward a behavior are determined by their beliefs and evaluations regarding the outcomes of that behavior [19].

Finally, intention to use (IU) refers to the degree to which users want to utilize a specific technology-related system for services, products, or tasks. In organizational settings, if members do not actively adopt new technologies, organizational efficiency may decline. Thus, the success of new technology adoption heavily relies on users’ acceptance and intention to use it [9]. Consequently, predicting the level of technology acceptance among organizational members before introducing new technology is a critical factor [15].

Considering that the average age of care workers in Korea is over 60, age is a significant factor in technology acceptance. The senior technology acceptance model (STAM) has been proposed for understanding technology acceptance among older adults [20]. This model incorporates factors such as gerontechnology anxiety (GA) and gerontechnology self-efficacy (GSE), considering the characteristics of older adults. Gerontechnology self-efficacy pertains to the confidence in one’s ability to effectively use gerontechnology, while gerontechnology anxiety describes the nervousness or fear an individual may feel when confronted with the prospect of using gerontechnology [20,21]. GSE positively relates to intention to use, whereas high gerontechnology anxiety tends to decrease intention to use [13,20].

Various researchers have expanded the TAM by incorporating additional factors that explain technology acceptance and use. Among these, the extended technology acceptance model (TAM 2) [22] includes subjective norms (SNs), defined as an individual’s perception of how others view their behavior. Additionally, the unified theory of acceptance and use of technology (UTAUT) introduced facilitating conditions (FCs), which denote the degree to which an individual believes that organizational and technological infrastructure supports the use of a technology [21].

Based on the above-mentioned factors, by actively adopting and utilizing the new technology of the robotic bed for repositioning, caregivers are anticipated to experience reduced physical strain, decreased incidence of musculoskeletal disorders, and enhanced job satisfaction. However, owing to the current lack of widespread adoption of care robots, few studies are available that report on the changes in caregivers’ perceptions of robots after their introduction, with research on care robots for older adults tending to focus on social robots [23,24,25,26]. Therefore, the empirical evidence on assistive technology for repositioning and preventing pressure ulcers, which are significant burdens on care workers, as well as the changes in technology acceptance among caregivers, needs to be examined.

Therefore, this study investigates whether the perception of caregivers working in nursing facilities in Korea significantly changes after using a robot product designed to prevent pressure ulcers and assist with repositioning.

## 2. Materials and Methods

### 2.1. Description of the Care Robot Bed to Prevent Pressure Sores

**Figure 1:**
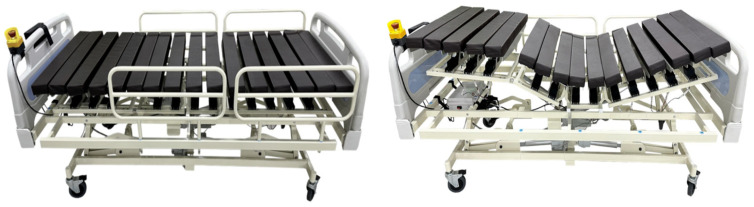
Smart care bed to prevent pressure sores.

This product is a combination medical device of the SCB-G model developed by Ninebell Healthcare and is a smart care bed equipped with a robotic system. The bed is ergonomically designed and allows for pressure control and position movement operated via a keyboard while the patient is lying down. All the built-in programs can be selected and operated using a remote control. Figure 1 below shows the product used in the test.

### 2.2. Participants

The recruitment process was conducted by three long-term care institutions located in Sungnam city, Gyeonggi-do, in Korea. The eligibility criteria for this study were as follows: (i) licensed care workers with one year or more experience, (ii) those without untreated serious or chronic internal medical diseases, (iii) those without musculoskeletal pain, and (iv) those without mental illness. All the participants provided written informed consent before taking part in the study.

Twenty long-term care workers were recruited across the three nursing facilities (W, E, J). Convenience sampling was used to recruit caregivers who wanted to use care robots. In addition, 94% of long-term care workers in Korea are women according to the 2023 Long-Term Care Insurance Statistical Yearbook; therefore, all the participants recruited for this study were women. Moreover, according to an OECD (2020) report [3], Korea has the highest average age for care workers among the OECD countries, with people in their 60s or above accounting for 65% of all care workers in Korea according to the Long-Term Care Survey [2]. The participants in this study also have similar characteristics to the general care industry in Korea. Among the participants in this study, two were in their 40s (10%), three were in their 50s (15%), and the rest were all in their 60s, accounting for 75% of the total participants. Based on previous studies showing that care workers’ physical pain increases as they age [4,27,28], the results of our participants’ age were somewhat higher than the Long-Term Care Survey [2], which indicates the needs of older care workers to participate in our experiments to reduce their physical burden since we recruited them voluntarily. Also, it shows the greater potential effect of care robots, given that the experiment was conducted with participants in these age groups.

The experiment was conducted in a living lab located in Seongnam city, the same region as the recruited care facilities, with 20 participants. The study, survey and recruitment were approved by the ethical committee of Kyung Hee University in Korea (KHGIRB-24-227-1). An overview of participants are shown in Table 1.

### 2.3. Experimental Protocol

The experimental protocol for this study consisted of three stages. In the first stage, a pre-survey was conducted in the living lab targeting long-term care workers. In the second stage, education and training were provided on how to use the robot and understand the device’s operation method and built-in programs. Since this was the participants first time experiencing smart care beds, we explained to them the usage of the smart care beds and the functions through a demonstration. Third, a position change demonstration was conducted using a dummy. In the last stage, a post-survey was conducted.

### 2.4. Surveys and Measurements

The participants completed a survey both before and after the experiment that included technology acceptance-related factors (PEOU, PE, AT, IU, FC, SN, gerontechnology anxiety, and self-efficacy) and demographic factors such as their age, gender, and care work experience. In accordance with the regulations set forth by the Kyung Hee University Institutional Review Board, all the data collected from research participants were anonymized to ensure privacy and prevent identification. An overview of the variables and their measurements and scales is shown in Table 2.

### 2.5. Data Analysis

The survey results from before and after experiencing the robot were converted into coded data and used for analysis. Frequency analysis and paired *t*-tests were used to analyze whether the participants’ acceptance of the technology changed after the demonstration relative to their acceptance before. Stata 17 was used for the analysis.

## 3. Results

### 3.1. Participant Demographics

All the participants were female, with an average age of 61.7 years. On average, they had been care workers for 68.65 months. Over 65% had graduated from high school, and 15% had graduated from college. More than 95% of the participants worked in two shifts. Table 3 provides the descriptive statistics for the participants.

### 3.2. Pre–Post Differences in Robot Use

#### 3.2.1. Perceived Usefulness

Table 4 shows that there were no significant differences in perceived usefulness in the overall average or subitems. This indicates that the short-term experiment did not provide sufficient insight into whether the robot could be useful for their intended tasks.

#### 3.2.2. Perceived Ease of Use (PEOU)

Both the overall average and all the subitems showed significant positive increases in perceived ease of use, as you can see in Table 5. This indicates that the participants found the method of using the robot to be very convenient and easy, leading to a perception change whereby they believed they could use it proficiently on their own.

#### 3.2.3. Attitude Toward Use (AT)

Table 6 shows that attitudes toward the robots slightly increased post-assessment compared to pre-assessment; however, no significant differences were found for the overall average or any subitems. Similar to intention to use, a positive intention toward future use was observed. However, in the current situation, significant differences were not immediately apparent.

#### 3.2.4. Intention to Use (IU)

The average intention to use robots did not show a significant difference; however, there was a positive inclination toward future robot use, as you can see in Table 7. Although two items showed a significant increasing trend, both were significant at *p* < 0.1, requiring careful interpretation.

#### 3.2.5. Gerontechnology Self-Efficacy (GSE)

As you can see in Table 8, self-efficacy did not show a significant change in the overall average; however, the perceived ability to use the robot well if provided with a manual showed a statistically significant increase, albeit marginally. This limited increase is likely because the participants’ overall self-efficacy scores were already quite high before the experiment. In Korea, long-term care workers receive extensive vocational training every two years, which includes pressure ulcer prevention [29]. Although the training is not specific to posture-changing robots, their experience of vocational training with various mattresses for pressure ulcer prevention [30] presumably contributed to their high existing self-efficacy. However, since the results for this variable were significant at *p* < 0.1, caution is needed when interpreting the results.

#### 3.2.6. Gerontechnology Anxiety (GA)

Anxiety also significantly decreased for both the overall average and all the subitems between before and after the experiment, as you can see in Table 9. Although the participants had some anxiety over using the robot before the experiment, their worries and fears about its use diminished afterwards.

#### 3.2.7. Subjective Norms (SNs)

Table 10 shows that both the overall average and all the subitems of subjective norms showed statistically significant increases, albeit marginally, after the experiment compared to before. This increase in subjective norms could be attributed to the participants’ involvement in the experiment. However, as the significance level was *p* < 0.1, caution is needed when interpreting these results.

#### 3.2.8. Facilitating Conditions (FCs)

The participants also showed positive changes before and after the experiment in terms of the availability, technological compatibility, and support provision for using robots, as you can in Table 11. The method for using the robots was not difficult, and the information learned through the experiment actively facilitated the conditions for using care robots. This led to positive perceptual changes in the relevant scale.

## 4. Discussion

To solve the problem of the increasing physical burden care workers experience because of aging and the shortage of care workers due to various disabilities, several countries are focusing on research on care robots. Japan invested in care robots early on, and Korea has also been investing heavily in full-scale care robots since 2019, especially the government. However, care robots are currently still not stably established in the field, including in Japan. The biggest reason for this is the mismatch between the functions of care robots as developed and the technologies required in the field [31,32,33].

Accordingly, experiments to verify the effectiveness of care robots in the care field have been receiving greater emphasis recently. When applying Korea’s care robots in the field, the improved physical health and reduced physical burden of care workers is important. However, for care robots to be used continuously in the field, care workers’ perceptions and attitudes must show positive changes. Although the initial adoption of robots may be ensured when they are deployed through governmental investment and support, the duration and extent of their subsequent utilization and procurement are contingent upon caregivers’ intentions once the governmental support is withdrawn.

This study examined whether the perceptions of robots significantly changed among care workers who had provided care to older adults for at least one year after they used robots designed to prevent pressure ulcers. Using paired *t*-tests, we gained significant insights into the use of robots in long-term care settings.

After using the robot, the participants showed changes in four TAM-related factors: perceived ease of use, subjective norms, and facilitating conditions were significant, and subjective norm showed a marginally significant difference. Furthermore, although the overall average was not significant, both intentions to use and gerontechnology self-efficacy showed some limited significant differences for the subitems. Considering that the change was after using the robot for a short period with 20 participants, the increase in perceived ease of use and subjective norms, coupled with the decrease in anxiety, suggests a reduction in resistance to performing caregiving tasks. Therefore, if the smart care bed is widely adopted in actual care facilities with long-term usage and systematic training, it could lead to positive attitudes among caregivers. When combined with other caregiving tasks, this could result in a significant reduction in workload and better outcomes.

The participants initially had a perception that the robot would be quite difficult to use, but after using the robot, their perception changed and they considered it easy to use. Therefore, the perceived ease of use of the care robot will likely be positive in the future. The results showed that perceived usefulness was not significant compared to perceived ease of use, indicating that the product was developed to be more user-friendly than expected and was not difficult to use; however, whether the robot will be helpful for their work remains uncertain. The smart care bed is designed to reduce the physical work required to change the position of bedridden patients to prevent bedsores. To do this, the device must be operated using a monitor with a panel and a remote control. The majority of the participants, who were in their 60s, may have had difficulty learning how to operate an unfamiliar robot, and this may not have led to a significant increase in the perceived usefulness. Previous studies have reported cases in which the usability of a technology in a workplace setting was negative owing to issues such as needing to learn a new robot/technology or adding work for maintenance of the robot or the new system. Barriers such as time constraints [34] or the need to learn how to operate new technology may burden users [35]. Although no such results were found for the robot in this study, efficient speed and minimized functions that can be useful in practice are necessary, in addition to functions of the robot that emphasize the ease of use.

Anxiety and self-efficacy in relation to robots are major variables for older adults. Considering the average age of Korean long-term care workers, efforts are needed to reduce gerontechnology anxiety and increase gerontechnology self-efficacy to improve their willingness to continuously use care robots. Care workers’ gerontechnology anxiety related to robots significantly decreased due to the robot being easier and more comfortable to use than expected. However, regarding gerontechnology self-efficacy, which reflects whether care workers can handle the robot well in practice, only some subitems showed limited significance. Whether care workers can use the robot well after using it for a short period showed limited change; however, this can be interpreted as indicating that it increased even during the short period of the experiment.

Education and training through continuous exposure to and experience with robots are important for increasing efficacy. In Japan, to increase the actual efficacy of robots, guides are provided for each robot, and robot experience centers are built nationwide to provide opportunities to interact with various robots in person. Korea is in the early stages of research on care robots; however, manuals for various care robots and short- and long-term education opportunities for care robot use are necessary to raise the overall awareness of robots before they are introduced in the aged care setting.

Increases in the subjective norms and facilitating conditions from the post-experiment suggest that social and environmental factors play crucial roles in technology acceptance. Positive changes in these areas indicate a supportive atmosphere for integrating robots, thereby highlighting the importance of comprehensive training and a supportive infrastructure.

This study has limitations, as it is inherently pre-experimental due to the current lack of widespread adoption of care robots. Additionally, it features a relatively small sample size of 20 participants, a single experimental design, and a short period of using the robot. Since experiences with robots may have different effects depending on the demographics and job characteristics of care workers, large-scale and long-term experiments are necessary. Considering that most participants have no prior experience of using care robots, it is essential to provide systematic education/training before starting the experiment. This education should include an explanation of how the robot can support long-term care workers in the caregiving workflow. Therefore, future research should include a group of at least 30 people for each experiment and a control group to confirm whether changes occur after using the robot for more than one month, including the education session before the engagement with the robots. In addition, this study only targeted the smart care bed to examine the effects of each robot; however, as various devices or equipment are used in actual care fields, research on various combinations of care robots according to the care services provided will also be necessary.

As an initial study for follow-up research, this study is meaningful in that it examined the changes in psychological perceptions among care workers who care for older adults in long-term care facilities. Considering the older age of Korean long-term care workers and the nature of their tasks, we anticipate that this study will contribute to strategies aimed at enhancing caregivers’ technology acceptance in relation to care robots and to establishing effective application methods.

## 5. Conclusions

The findings of this study demonstrated that care workers’ perceptions shifted significantly following the integration of care robots. This underscores the critical factors that must be prioritized to foster positive perceptions and attitude changes among care workers toward the utilization of care robots. These insights can form a foundational basis for the development and enhancement of care robots tailored to the specific needs of care environments, as well as for the formulation of effective implementation policies within care settings. It is also important to note that care technologies, such as the smart care bed used in this study, are intended to supplement care work by reducing the workload of long-term care workers and improving efficiency, not to replace in-person services entirely.

## Figures and Tables

**Table 1:** Participant characteristics (N = 20).

Participant	Age	Gender	Working Period (Months)	Institution
A	62	Female	23	W
B	57	Female	62	W
C	63	Female	108	W
D	56	Female	134	W
E	62	Female	1	E
F	63	Female	123	E
G	47	Female	42	J
H	57	Female	48	J
I	65	Female	60	E
J	48	Female	60	W
K	68	Female	20	J
L	61	Female	108	E
M	67	Female	120	J
N	65	Female	13	W
O	68	Female	75	E
P	66	Female	108	W
Q	64	Female	108	J
R	69	Female	16	J
S	66	Female	24	E
T	60	Female	120	J

**Table 2:** Variables, measurements, and scales used in the study.

Measurements and Scales			
TAM- related factors	Perceived usefulness (PU)	3 items, strongly disagree (1)~strongly agree (5)	Davis [9]
	Perceived ease of use (PEOU)	2 items, strongly disagree (1)~strongly agree (5)	
	Attitude (AT)	2 items, strongly disagree (1)~strongly agree (5)	
	Intention to use (IU)	3 items, strongly disagree (1)~strongly agree (5)	
STAM- related factors	Gerontechnology self-efficacy (GSE)	2 items, strongly disagree (1)~strongly agree (5)	Chen and Chan [20]
	Gerontechnology anxiety (GA)	2 items, strongly disagree (1)~strongly agree (5)	
Expanded-TAM- related factors	Subjective norms (SN)	2 items, strongly disagree (1)~strongly agree (5)	Davis and Ventakesh [22]
	Facilitating conditions (FC)	3 items, strongly disagree (1)~strongly agree (5)	

**Table 3:** Descriptive statistics (N = 20).

Variables	Values	Total	
		Freq. (n)/Mean	%/SD
Gender	Female	20	100
Age (years)	(Mean)	61.7	6.12
	≤54	2	10.0
	55–59	3	15.0
	60–64	6	30.0
	≥65	9	45.0
Working period as care worker (months)		68.65	44.10
Education level	~Middle school	4	20.0
	High school	13	65.0
	College~	3	15.0
Workplace	W nursing home	7	35.0
	E nursing home	6	30.0
	J nursing home	7	35.0
Working shift	Two shifts	19	95.0
	Three shifts	1	5.0
Personal Income	(Mean, KRW 10,000)	340.55	393.92

**Table 4:** Pre–post differences in perceived usefulness (N = 20).

Variables	Pre	Post	Difference	t	*p*
Overall mean	3.7	3.81	−0.11	−0.79	0.4392
Item 1 ^1^	3.7	3.85	−0.15	−1.0	0.3299
Item 2 ^2^	3.7	3.8	−0.1	−0.62	0.5409
Item 3 ^3^	3.7	3.8	−0.1	−0.62	0.5409

^1^ Item 1: Using technology would enhance your effectiveness in life; ^2^ Item 2: Using technology would make your life more convenient; ^3^ Item 3: You would find technology useful for performing your tasks.

**Table 5:** Pre–post differences in perceived ease of use (N = 20).

Variables	Pre	Post	Difference	t	*p*
Overall mean	3.17	3.8	−0.625	−5.78	0.0000
Item 1 ^1^	3.15	3.75	−0.6	−3.94	0.0009
Item 2 ^2^	3.2	3.85	−0.65	−5.94	0.0000

^1^ Item 1: You would find the technology easy to use; ^2^ Item 2: You could use the technology skillfully.

**Table 6:** Pre–post differences in attitude toward use (N = 20).

Variables	Pre	Post	Difference	t	*p*
Overall mean	3.7	3.9	−0.2	−1.7097	0.1036
Item 1 ^1^	3.7	3.9	−0.2	−1.7	0.1036
Item 2 ^2^	3.7	3.9	−0.2	−1.7	0.1036

^1^ Item 1: Using technology is a good idea; ^2^ Item 2: You like the idea of using technology.

**Table 7:** Pre–post differences in intention to use (N = 20).

Variables	Pre	Post	Difference	t	*p*
Overall mean	3.61	3.83	−0.21	−1.68	0.1084
Item 1 ^1^	3.7	3.8	−0.1	−0.69	0.4936
Item 2 ^2^	3.6	3.85	−0.25	−1.75	0.0961
Item 3 ^3^	3.55	3.85	−0.3	−2.04	0.0553

^1^ Item 1: I intend to use the robot in the future; ^2^ Item 2: I predict I will use the robot in the future; ^3^ Item 3: I plan to use the robot in the future.

**Table 8:** Pre–post differences in gerontechnology self-efficacy (N = 20).

Variables	Pre	Post	Difference	t	*p*
Overall mean	3.77	3.97	−0.2	−1.70	0.1036
Item 1 ^1^	3.85	4	−0.15	−1.0	0.3299
Item 2 ^2^	3.7	3.95	−0.25	−2.03	0.0563

^1^ Item 1: You could complete a task using the robot if someone demonstrates how; ^2^ Item 2: You could complete a task using the robot if you have only the instruction manual for assistance.

**Table 9:** Pre–post differences in gerontechnology anxiety (N = 20).

Variables	Pre	Post	Difference	t	*p*
Overall mean	2.325	1.925	0.4	2.3196	0.0317
Item 1 ^1^	2.4	1.95	0.45	2.01	0.0583
Item 2 ^2^	2.25	1.9	0.35	2.33	0.0308

^1^ Item 1: You feel apprehensive about using the robot; ^2^ Item 2: You hesitate to use the robot for fear of making mistakes you cannot correct.

**Table 10:** Pre–post differences in subjective norms (N = 20).

Variables	Pre	Post	Difference	t	*p*
Overall mean	3.15	3.52	−0.37	1.92	0.0695
Item 1 ^1^	3.15	3.5	−0.35	−1.92	0.0692
Item 2 ^2^	3.15	3.55	−0.4	−1.79	0.0880

^1^ Item 1: People who influence my behavior think that I should use the robot; ^2^ Item 2: People who are important to me think that I should use the robot.

**Table 11:** Pre–post differences in facilitating conditions (N = 20).

Variables	Pre	Post	Difference	t	*p*
Overall mean	2.86	3.45	−0.58	−3.19	0.0047
Item 1 ^1^	2.85	3.65	−0.8	−4.29	0.0004
Item 2 ^2^	2.85	3.35	−0.5	−2.70	0.0141
Item 3 ^3^	2.9	3.35	−0.45	−2.13	0.0464

^1^ Item 1: You have the knowledge necessary to use the robot; ^2^ Item 2: You can use the robot compatible with other devices; ^3^ Item 3: A specific person (or group) is available to assist with technology difficulties.

## Data Availability

The data are not publicly available due to restrictions (they contain information that could compromise the privacy of research participants).
